# Author Correction: Lin28 and *let-7* regulate the timing of cessation of murine nephrogenesis

**DOI:** 10.1038/s41467-020-14944-3

**Published:** 2020-03-09

**Authors:** Alena V. Yermalovich, Jihan K. Osborne, Patricia Sousa, Areum Han, Melissa A. Kinney, Michael J. Chen, Daisy A. Robinton, Helen Montie, Dan S. Pearson, Sean B. Wilson, Alexander N. Combes, Melissa H. Little, George Q. Daley

**Affiliations:** 1 Division of Pediatric Hematology/Oncology, Children’s Hospital Boston, Boston, MA 02115 USA; 2 Department of Biological Chemistry and Molecular Pharmacology, Harvard Medical School, Boston, MA 02115 USA; 3 Harvard Stem Cell Institute, Boston, MA 02115 USA; 4 Murdoch Childrens Research Institute, Royal Children’s Hospital, 50 Flemington Rd, Parkville, Melbourne, 3052 Australia; 5 Department of Anatomy and Neuroscience, Faculty of Science, Building 181, Corner of Grattan Street and Royal Parade, University of Melbourne, Parkville, 3010 Australia; 6 Department of Pediatrics, The University of Melbourne, Level 2 West, The Royal Children’s Hospital, 50 Flemington Road, Parkville, 3010 Australia

**Keywords:** Developmental biology, Embryogenesis, Nephrons

Correction to: *Nature Communications* 10.1038/s41467-018-08127-4, published online 11 January 2019.

The original version of this Article contained an error in Fig. 2b, in which the P21 LIN28B Wt1 panel was inadvertently duplicated and the same panel used for the P14 image in error. The correct version of Fig. 2b is:



which replaces the previous incorrect version:



This has been corrected in both the PDF and HTML versions of the Article.

